# Selection effects may account for better outcomes of the German Disease Management Program for type 2 diabetes

**DOI:** 10.1186/1472-6963-10-351

**Published:** 2010-12-31

**Authors:** Ingmar Schäfer, Claudia Küver, Benjamin Gedrose, Eike-Christin von Leitner, András Treszl, Karl Wegscheider, Hendrik van den Bussche, Hanna Kaduszkiewicz

**Affiliations:** 1 Department of Primary Medical Care, Center of Psychosocial Medicine, University Medical Center Hamburg-Eppendorf, Martinistr. 52, 20246 Hamburg, Germany; 2 Department of Medical Biometry and Epidemiology, Center for Experimental Medicine, University Medical Center Hamburg-Eppendorf, Martinistr. 52, 20246 Hamburg, Germany

## Abstract

**Background:**

The nationwide German disease management program (DMP) for type 2 diabetes was introduced in 2003. Meanwhile, results from evaluation studies were published, but possible baseline differences between DMP and usual-care patients have not been examined. The objective of our study was therefore to find out if patient characteristics as socio-demographic variables, cardiovascular risk profile or motivation for life style changes influence the chance of being enrolled in the German DMP for type 2 diabetes and may therefore account for outcome differences between DMP and usual-care patients.

**Methods:**

Case control study comparing DMP patients with usual-care patients at baseline and follow up; mean follow-up period of 36 ± 14 months. We used chart review data from 51 GP surgeries. Participants were 586 DMP and 250 usual-care patients with type 2 diabetes randomly selected by chart registry. Data were analysed by multivariate logistic and linear regression analyses. Significance levels were p ≤ 0.05.

**Results:**

There was a better chance for enrolment if patients a) had a lower risk status for diabetes complications, i.e. non-smoking (odds ratio of 1.97, 95% confidence interval of 1.11 to 3.48) and lower systolic blood pressure (1.79 for 120 mmHg vs. 160 mmHg, 1.15 to 2.81); b) had higher activity rates, i.e. were practicing blood glucose self-monitoring (1.67, 1.03 to 2.76) and had been prescribed a diabetes patient education before enrolment (2.32, 1.29 to 4.19) c) were treated with oral medication (2.17, 1.35 to 3.49) and d) had a higher GP-rated motivation for diabetes education (4.55 for high motivation vs. low motivation, 2.21 to 9.36).

**Conclusions:**

At baseline, future DMP patients had a lower risk for diabetes complications, were treated more intensively and were more active and motivated in managing their disease than usual-care patients. This finding a) points to the problem that the German DMP may not reach the higher risk patients and b) selection bias may impair the assessment of differences in outcome quality between enrolled and usual-care patients. Suggestions for dealing with this bias in evaluation studies are being made.

## Background

Sufficient evaluation is a precondition for deciding whether the implementation of any new health care policy was successful. Policy makers depend on this scientific evidence, but their decisions sometimes restrain researchers from generating evidence in a valid way. This is the case with the nationwide disease management program (DMP) for type 2 diabetes, which was introduced in Germany in 2003.

The DMP was started in a rush with no preceding randomized controlled trial to evaluate its effectiveness. Instead, the program immediately became a regular part of the statutory health care system, which covers 86% of the German population [1]. This decision prohibited a proper evaluation of its effects as we will describe later. Nevertheless, researchers have designed several evaluation projects and recent publications suggest that the German DMP is a highly effective intervention: DMP patients are supposed to have fewer and shorter hospital stays as well as less microvascular and cardiovascular complications [2]. Enrolment in the DMP is also supposed to lead to a better health-related quality of life [3] and may even result in a significant reduction in mortality compared to care as usual [4]. But these conclusions may be premature, as the studies did not consider selection effects regarding the inclusion of patients in the DMP.

A comprehensive description of the history and design of the German DMP is found in another paper [5]. In short, the DMP is a complex intervention including different medical services, e.g. regular foot and eye examinations, rules for referrals to specialists and participation in diabetes education courses. Enrolment in the DMP is voluntary for physicians and patients, but endorsed with financial incentives for both. According to latest data, in August 2009 approximately 64% of the estimated five million statutory insured patients with type 2 diabetes were enrolled in the program [6].

The population-based approach of the German DMP is somewhat restricted as the law states that only those patients should be included who will participate actively and are expected to benefit from the program [7]. It is up to the attending physicians to decide if and how they apply these enrolment criteria. However, due to these criteria it is conceivable that patients are not enrolled in the DMP if their doctors consider them to be inactive or rather non-compliant [8].

For health services research on DMP effectiveness this implies that there may be baseline differences between DMP and usual-care patients influencing the treatment of their diabetes or their prognosis. Therefore non-randomized observational studies may suffer from a selection bias and differences in process and outcome quality may result from different patient characteristics in the two groups instead of program elements. For that reason the question whether such a selection bias regarding enrolment in the DMP exists is crucial for the interpretation of study results [9].

The objective of our study was therefore to find out if patient characteristics as socio-demographic variables, cardiovascular risk profile or motivation for life style changes influence the chance of being enrolled in the German DMP for type 2 diabetes and to explore the impact of this possible selection mechanism on DMP effectiveness and the assessment of DMP outcome measures.

## Methods

The study is designed as a case-control study in which DMP patients are compared with usual-care patients. We collected present data and performed a retrospective baseline assessment at the dates the DMP patients had been enrolled. We chose the 30^th ^of June 2005 as the index date for the selection of usual-care patients, because it was estimated as mean date of enrolment of the DMP patients. In the following we will refer to present data as "t_1_" and baseline data as "t_0_". The study was approved by the Ethics Committee of the Medical Association of Hamburg (reference number OB-008/07).

We randomly selected patients via chart registry from 51 primary care surgeries located in and around 4 larger German cities (Hamburg, Bremen, Berlin and Düsseldorf). In each surgery we created a list of eligible patients based on the electronic database of the GP. To be eligible for the study the patients had to be treated for type 2 diabetes at least since June 2005 by their GP and they had to have visited the GP surgery at least once in the last 12 months. Patients were excluded if they were no regular patients of the participating surgery, if they had severe medical problems seriously affecting their ability to participate in the study or if they were enrolled in the DMP for shorter than 6 months at t_1_. From all eligible patients we randomly selected a maximum of 25 DMP patients and 25 usual-care patients and contacted them for written informed consent to data collection based on the documentation of their GP. However, since DMP participation rates were above 50% in most of the participating surgeries, the number of eligible usual-care patients was smaller than scheduled.

**Figure 1:**
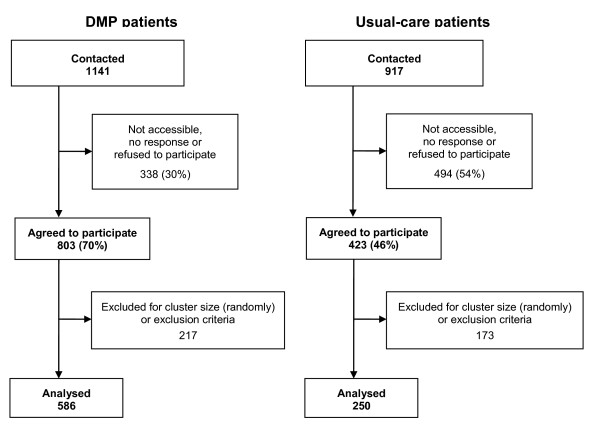
**Sampling and response rate**.

Altogether, 1141 DMP-patients and 917 usual-care patients were contacted (cf. Figure 1). 60% of all contacted patients (70% DMP and 46% usual-care patients) agreed to participate in the study. Budget constraints forced us to restrict the cluster size further to be able to retain a high number of surgeries. For that reason we randomly selected from all patients who agreed to participate a maximum of 15 DMP and 15 usual-care patients per surgery. In sum 217 DMP patients and 173 usual-care patients were excluded for cluster size restrictions or because we found out in plausibility checks that they complied with exclusion criteria, i.e. DMP patients being enrolled for less than 6 months or usual-care patients with no diagnosis of diabetes at t_0_. In total, 586 DMP patients and 250 usual-care patients were eligible for inclusion. Recruitment and data collection took place from 10^th ^of July 2007 to 16^th ^of June 2009.

### Data collection

Data collection techniques included chart review and interviews with GPs. For chart review the interviewers used the GPs' electronic and/or paper records and the official DMP documentation sheets. For transferring the collected data we used double data entry and additional plausibility checks during and after data entry.

The data collected from chart review encompassed age, gender, height, weight, duration of diabetes, co-morbidity (e.g. depression and hypertension), smoking behaviour, laboratory data of glycated haemoglobin (GHb) values, blood pressure (BP) values, diabetic symptoms (polyuria, polydipsia, fatigue), diabetes medication (insulin and/or oral medication), glucose self-monitoring, and documented prescriptions for a diabetes patient education course. For a proxy assessment of the patients' general motivation regarding life style changes we asked the GPs to rate the patients' motivation for diabetes education on a 3 point scale (low, medium, high). Additionally, we asked the GPs if their surgeries offered diabetes patient education. We used this variable as a proxy measure for the GPs' knowledge and activity regarding diabetes.

### Missing data

The data file included a certain amount of missing values. Although 16 of 21 variables had less than 10% missing values (cf. table 1), we identified a total of 357 complete and 489 incomplete data sets. Complete and incomplete data sets differed significantly regarding enrolment status in the sense that DMP patients were more likely to have complete data sets (cf. table 2). This phenomenon is a result of the GPs' inferior quality of patient documentation in usual-care patients outweighing our efforts to include as much information as possible.

**Table 1:** Missing cases in available data

	t_0_			t_1_		
Variable	total (n = 836)	usual-care (n = 250)	DMP (n = 586)	total (n = 836)	usual-care (n = 250)	DMP (n = 586)
Duration of diabetes	not applicable			**5.2%**	12.8%	2.1%
Co-morbidity: hypertension	not determined			**1.8%**	1.2%	2.1%
Co-morbidity: depression	not determined			**1.8%**	1.2%	2.1%
Smoking behaviour	**10.3%**	20.4%	6.1%	**11.6%**	18.4%	8.7%
GHb values	**3.8%**	10.4%	0.9%	**2.7%**	8.8%	0.2%
BP values	**5.6%**	16.8%	0.9%	**3.9%**	8.8%	1.9%
Diabetic symptoms	**9.2%**	18.4%	5.5%	**7.9%**	12.0%	6.3%
Oral medication	**2.5%**	5.6%	1.2%	**0.9%**	1.2%	0.9%
Insulin treatment	**2.5%**	5.6%	1.2%	**1.1%**	1.2%	1.0%
Glucose self-monitoring	**7.6%**	10.4%	6.5%	**4.5%**	4.4%	4.6%
Previous prescription of a patient education program	**15.7%**	22.4%	13.0%	**9.8%**	12.4%	8.9%
GP-rated motivation of the patients	**20.7%**	24.4%	19.3%	**14.1%**	13.6%	14.3%

**Table 2:** Comparison of complete and incomplete data sets (t-test/χ2-test)

Variable	complete (n = 357)	incomplete (n = 489)	p
Age [in years]: mean ± standard deviation (SD)	69.3 ± 10.4	69.5 ± 11.5	n.s.
Women in %	47.3%	47.2%	n.s.
DMP patients in %	78.7%	63.4%	< 0.001
Duration of diabetes [in months]: mean ± SD	115 ± 86	110 ± 78	n.s.
GHb [in %]: mean ± SD	7.0 ± 1.1	6.9 ± 1.1	n.s.
Systolic BP [in mmHg]: mean ± SD	137 ± 17	139 ± 17	n.s.
Diastolic BP [in mmHg]: mean ± SD	79 ± 9	79 ± 10	n.s.
Motivation [3 = high through 1 = low]: mean ± SD	1.6 ± 0.8	1.6 ± 0.8	n.s.

n.s.: statistically not significant (p > 0.05)

To reduce the possibility of misleading results based on the distribution of missing data, we decided to impute missing values by multiple imputation, which is considered to be the statistically most valid imputation method [10]. It has been argued that for research on progressive diseases like type 2 diabetes, not more than 20% of missing values can be tolerated [11]. We therefore excluded all variables from the analyses that had a total proportion of missing values of 21% and more.

In the imputation process, we created M = 100 data sets with multivariate normal regression. We used a total run length of 100,000 iterations with imputations made after every 1,000^th ^iteration. We performed log transformations to deal with non-normality of continuous variables. Complete data were transformed back to their original scales before analysis. We transformed categorical variables to dummy variables and refitted them to their scale after imputation [12].

The imputation model included all variables from the complete data model including variables that could have affected the response (e.g. depression) and additional variables that were highly correlated (i.e. frequency of GHb and BP measurements, diabetic symptoms, hypertension as co-morbidity and body mass index). Age, gender and enrolment status were used for the imputation model, but not imputed themselves.

### Statistical analyses

We analysed differences in baseline characteristics between DMP and usual-care patients by bivariate t-tests and χ^2^-tests. The existence and the direction of a selection bias were assessed using multivariate logistic regression: DMP and usual-care patients were compared at baseline regarding socio-demographic data (age and gender), morbidity (depression and duration of diabetes), risk status for diabetic complications (smoking behaviour, GHb and systolic BP values), treatment intensity (medication), patient self-activity (earlier prescriptions of a diabetes education program as well as urinary and blood glucose self-monitoring) and the GP-rated motivation of the patients to participate in a diabetes education program. All factors were analysed in a unitary statistical model and were therefore in each case adjusted for the influence of the other variables. Additionally, the analysis was controlled for diabetes education activity of the GP.

Concerning the impact of the selection bias, outcome indicators were analysed by multivariate OLS regression taking the observation periods between t_0 _and t_1 _into account. To assess the outcome quality, we analysed the course of GHb and systolic BP values in relation to baseline values. Age, gender, duration of diabetes, depression, medication (regarding GHb only), smoking status (regarding BP only) and diabetes education activity of the GP were used as control variables.

To estimate the selection bias due to drop out we performed 'quasi'-intention to treat analyses for the assessment of selection bias and outcome quality by treating usual-care patients as DMP patients if they had ever been enrolled in the DMP in the past. These analyses used the same statistical modelling as described above.

All multivariate regression analyses accounted for stratification of GP surgeries in four different cities and reduced variance because of patient clustering in GP surgeries. For all analyses an α-level of 5% (p ≤ 0.05) was defined as statistically significant. Data analyses and imputations of missing values were performed using Stata 11.

We performed a power calculation for the outcome analyses to ascertain that the final sample size (586 DMP patients and 250 usual-care patients) was sufficient. Means and standard deviations of GHb (6.9 ± 1.2%) and systolic BP (140.6 ± 18.3 mmHg) were derived from the German DETECT study [13]. To assess the cluster effect we followed recommendations to assume an intra class correlation of 0.04 in general practice [14]. We estimated a design effect of 1.64 using the existing distribution of patients in surgeries. Following results from the UKPDS study, we defined 1% in GHb [15] and 10 mmHg in systolic BP [16] as clinically relevant differences. Statistical power (1-β) for outcome differences in both GHb and systolic BP was > 99% considering the existing sample size.

## Results

The analysis is based on 586 DMP patients and 250 usual-care patients. The average observation period between t_0 _and t_1 _was 34 ± 17 months for DMP patients and 40 ± 5 months for usual-care patients, respectively. Due to the study design (case-control study with retrospective baseline) the calendar date of t_0 _varies for the study participants, i.e. the factual date of enrolment for DMP patients and 30^th ^of June 2005 for usual-care patients. Usual-care patients were significantly older at baseline (67.4 ± 12.5 years vs. 66.0 ± 10.3 years) and had a shorter duration of illness than DMP patients (5.2 ± 5.5 years vs. 6.8 ± 7.2 years). 52.6% of the study participants were male. We found no gender differences between DMP patients and usual-care patients. Differences in baseline characteristics between DMP and usual-care patients are shown in table 3.

**Table 3:** Differences in baseline characteristics between DMP and usual-care patients

Variable	usual-care patients (n = 250)	DMP patients (n = 586)	p*
***Age (in years): mean ± standard deviation (SD)***	***67.4 ± 12.5*** ***(n = 250)***	***66.0 ± 10.3*** ***(n = 586)***	***0.041***
Gender: men	128 (51.2%)	312 (53.2%)	0.588
***Duration of diabetes (in years) ± SD***	***5.2 ± 5.5*** ***(n = 218)***	***6.8 ± 7.2*** ***(n = 574)***	***0.016***
Co-morbidity: depression	28 (11.3%)	52 (9.1%)	0.313
Smoking behaviour: non-smoking	157 (78.9%)	466 (84.7%)	0.059
GHb value (in %) ± SD	6.9 ± 1.2 (n = 224)	7.0 ± 1.1 (n = 581)	0.153
***Systolic BP value (in mmHg) ± SD***	***144 ± 23*** ***(n = 208)***	***139 ± 17*** ***(n = 581)***	***0.002***
***Oral medication***	***119 (50.4%)***	***384 (66.3%)***	***< 0.001***
Insulin dependency	32 (13.6%)	90 (15.5%)	0.471
***Blood glucose self-monitoring***	***45 (20.1%)***	***158 (28.8%)***	***0.012***
***Urinary glucose self-monitoring***	***5 (2.2%)***	***55 (10.4%)***	***< 0.001***
***Prescription of diabetes education before DMP start***	***25 (12.9%)***	***206 (40.4%)***	***< 0.001***
***GP-rated motivation of the patients***	***low: 148 (78.3%)*** ***medium: 23 (12.2%)*** ***high: 18 (9.5%)***	***low: 199 (42.1%)*** ***medium: 156 (33.0%)*** ***high: 118 (25.0%)***	***< 0.001***

* Statistically significant results (p ≤ 0.05) in italic and bold letters.

### Assessment of selection bias

In the logistic regression analysis we found that many factors were associated with enrolment in the DMP for type 2 diabetes (cf. table 4). Patients with a lower risk for diabetic complications had a better chance of being enrolled, i.e. non-smokers (odds ratio 2.0) and patients with lower systolic BP (1.8 for 120 mmHg vs. 160 mmHg). Furthermore, treatment with oral medication (2.2) increased the likeliness of enrolment. Other factors contributing to a higher probability of enrolment were higher self-activity rates, i.e. performing blood glucose self-monitoring (1.7) and having been prescribed a diabetes education program (2.3). The most important predictor for enrolment was the GP-rated motivation of the patients to participate in a diabetes education program (4.6 for high motivation vs. low motivation). Despite the fact that bivariate analyses pointed at significant differences in age, urinary glucose self-monitoring and diabetes duration (see above), these variables did not affect the chance for enrolment in the multivariate analysis after adjusting for possible confounders.

**Table 4:** Factors associated with future enrolment in the DMP. Results from logistic regression analysis (n = 836)

Variable	Odds ratio	95% confidence interval	p*
Age: 60 vs. 70 years	1.12	0.91 to 1.37	0.254
Gender: men	1.09	0.71 to 1.68	0.681
Duration of diabetes: 10 years vs. 1 year	1.02	0.99 to 1.05	0.125
Co-morbidity: no depression	1.33	0.80 to 2.21	0.264
***Smoking behaviour: non-smoking***	***1.97***	***1.11 to 3.48***	***0.021***
GHb value: 7.0 vs. 8.0%	1.04	0.88 to 1.23	0.624
***Systolic BP value: 120 vs. 160 mmHg***	***1.79***	***1.15 to 2.81***	***0.012***
Oral medication**	***2.17***	***1.35 to 3.49***	***0.002***
***Blood glucose self-monitoring***	***1.69***	***1.03 to 2.76***	***0.038***
Urinary glucose self-monitoring	2.96	0.92 to 9.47	0.067
***Prescription of diabetes education program before DMP start***	***2.32***	***1.29 to 4.19***	***0.006***
***GP-rated motivation of the patients: high vs. low (3 vs. 1 on a 3-point scale)***	***4.55***	***2.21 to 9.36***	***< 0.001***
Diabetes education activity of the GP	1.23	0.71 to 2.14	0.456

* Statistically significant results (p ≤ 0.05) in italic and bold letters.

** Insulin treatment was dropped from the statistical model in favour of blood glucose self-monitoring because of high correlation between these two variables (r^2 ^> 0.5) and blood glucose self-monitoring being the better predictor for enrolment.

### Outcome quality

We chose the course of GHb and systolic BP values between t_0 _and t_1 _as indicators of outcome quality (cf. table 5). We found a significant difference in the course of systolic BP between DMP-patients and usual-care patients. DMP patients had a statistically significant lower systolic BP at baseline (139 mmHg vs. 144 mmHg for usual-care patients) and DMP patients also showed a greater decrease in systolic BP between t_0 _and t_1_, that was 3.6 mmHg higher than for usual-care patients. The course of systolic BP was also influenced by the duration of diabetes (i.e. +0.2 mmHg for every year of diabetes).

**Table 5:** Comparison of outcome quality in DMP and usual-care. Results from multivariate linear regression analyses (n = 836)

Outcome indicators	DMP versus usual-care patients (adjusted)	95% confidence interval	p*
Course of GHb	-0.11%	-0.63 to 0.28	0.208
***Course of systolic BP***	***-3.57 mmHg***	***-0.98 to -6.15***	***0.008***

Adjusted for length of observation period between t0 and t1, age, gender, duration of diabetes, depression, medication (regarding GHb only), smoking status (regarding BP only) and diabetes education activity of the GP.

* Statistically significant results (p ≤ 0.05) in italic and bold letters.

GHb at baseline was 6.9 ± 1.1% for both groups. There was no change between t_0 _and t_1 _(i.e. 0.0 ± 1.1%). We found no statistical association between the course of GHb and enrolment in the DMP. Independent of the enrolment status the course of GHb values over time was significantly associated with the patients' medication (i.e. insulin treatment: +0.6%, oral medication +0.3%).

### Comparison of available and imputed data sets

We compared the results of all analyses when based on imputed data with those when based on available data. There were considerable differences between the results based on imputed compared to available data (cf. table 6), however, the direction of the associations stayed the same.

**Table 6:** Statistically significant differences between DMP and usual-care patients when using imputed and available data

	Imputed data	Available data
**Selection bias**	1. Systolic BP value: 120 vs. 160 mmHg (OR: 1.79 [95% CI: 1.15 to 2.81])	1. Systolic BP value: 120 vs. 160 mmHg (OR: 1.99 [95% CI: 1.09 to 3.63])
	2. Oral medication (OR: 2.17 [95% CI: 1.35 to 3.49])	2. Oral medication (OR: 2.32 [95% CI: 1.28 to 4.20])
	3. Blood glucose self-monitoring (OR: 1.69 [95% CI: 1.03 to 2.76])	3. Blood glucose self-monitoring (OR: 3.40 [95% CI: 1.70 to 6.80])
	4. Prescription of diabetes patient education (OR: 2.32 [95% CI: 1.29 to 4.19])	4. Prescription of diabetes patient education (OR: 2.64 [95% CI: 1.27 to 5.41])
	5. GP-rated motivation of the patients: high vs. low (3 vs. 1 on a 3-point scale) (OR: 4.55 [95% CI: 2.21 to 9.36])	5. GP-rated motivation of the patients: high vs. low (3 vs. 1 on a 3-point scale) (OR: 6.96 [95% CI: 2.26 to 21.43])
	6. Smoking behaviour: non-smoking (OR: 1.97 [95% CI: 1.11 to 3.48])	6. No difference regarding smoking behaviour
	7. No difference regarding urinary glucose self-monitoring	7. Urinary glucose self-monitoring (OR: 5.68 [95% CI: 1.07 to 30.21])
		
**Outcome quality**	1. Course of systolic BP (-3.57 mmHg [95% CI: -0.98 to -6.15])	1. No differences regarding course of systolic BP

OR: odds ratio; CI: confidence interval

### Quasi-intention-to-treat-analysis

We found 21 usual-care patients who temporarily had been enrolled in the DMP before the study started. If we count these patients as DMP patients, the results from our analysis are only marginally different from the results presented in this paper (cf. table 7).

**Table 7:** Statistically significant differences between DMP and usual-care patients when using original and quasi-intention-to-treat analyses sets

	Original analyses	Quasi-intention-to-treat analyses
**Selection bias**	1. Systolic BP value: 120 vs. 160 mmHg (OR: 1.79 [95% CI: 1.15 to 2.81])	1. Systolic BP value: 120 vs. 160 mmHg (OR: 1.95 [95% CI: 1.22 to 3.11])
	2. Oral medication (OR: 2.17 [95% CI: 1.35 to 3.49])	2. Oral medication (OR: 2.60 [95% CI: 1.62 to 4.17])
	3. Blood glucose self-monitoring (OR: 1.69 [95% CI: 1.03 to 2.76])	3. Blood glucose self-monitoring (OR: 1.83 [95% CI: 1.08 to 3.11])
	4. Prescription of diabetes patient education (OR: 2.32 [95% CI: 1.29 to 4.19])	4. Prescription of diabetes patient education (OR: 2.88 [95% CI: 1.48 to 5.59])
	5. GP-rated motivation of the patients: high vs. low (3 vs. 1 on a 3-point scale) (OR: 4.55 [95% CI: 2.21 to 9.36])	5. GP-rated motivation of the patients: high vs. low (3 vs. 1 on a 3-point scale) (OR: 4.64 [95% CI: 2.04 to 10.53])
	6. Smoking behaviour: non-smoking (OR: 1.97 [95% CI: 1.11 to 3.48])	6. No difference regarding smoking behaviour
	7. No difference regarding urinary glucose self-monitoring	7. Urinary glucose self-monitoring (OR: 3.78 [95% CI: 1.13 to 12.61])
		
**Outcome quality**	1. Course of systolic BP (-3.57 mmHg [95% CI: -0.98 to -6.15])	1. Course of systolic BP (-3.31 mmHg [95% CI: -1.01 to -5.63])

OR: odds ratio; CI: confidence interval

## Discussion

We found that before enrolment future DMP-patients differed from usual-care patients in four dimensions. Patients were more likely to be enrolled into the DMP if they

1. had a lower risk status for diabetic complications (i.e. non-smoking and lower systolic BP),

2. had higher self-activity rates (i.e. blood glucose self-monitoring and having been prescribed a diabetes education program prior to the DMP),

3. were treated with oral medication and

4. had a higher GP-rated motivation regarding participation in diabetes patient education.

All these dimensions are generally considered to have a strong positive influence on treatment effects and on the prognosis of diabetes.

We compared the chances for enrolment by odds ratios, which are standard measures for these analyses. Odds ratios are less intuitionally understood than risk ratios because they are based on differences in odds, not in likelihood as risk ratios (e.g. a number on a die has a 1/5 odds, but a 1/6 likelihood). An odds ratio of 4.55 for GP-rated high motivation vs. low motivation of the patients means that a patient with high motivation has 4.55-times higher odds for enrolment than a person with low motivation.

If exposure variables have a low prevalence (i.e. 5% and below) odds ratios and risk ratios are comparable. As the factors we identified in this study are common (prevalence > 10%), the odds ratios (OR) presented in table 4 do not approximate risk ratios (RR). It is possible to convert OR into RR by accounting for the frequency of each factor [17].

In doing this the 4.55 odds ratio (with a total prevalence of high motivation of 20.54% in the sample) translates to a 2.63 risk ratio. This means that a highly motivated patient has 2.63-times higher likelihood for enrolment than a person having a low motivation. As we regard these differences still as highly clinically relevant, we conclude that a selection bias has to be taken into account when evaluating process and/or outcome results of the DMP for type 2 diabetes in Germany. However, the possibility of statistical adjustment for this selection bias is limited, as we will discuss later on.

It is important to decide if the bias found in our study mainly results from discontinuation of DMP enrolment (drop out) [18] or from the GPs selection mechanisms in clinical reality. Case-control studies (and all other studies recruiting patients after the intervention started) may suffer from a selection bias occurring when patients systematically drop out of the intervention group before data collection (e.g. because of low motivation or severe illness). Therefore we examined this possibility in our study by a 'quasi'-intention to treat analysis counting usual-care patients as DMP patients if they had ever been enrolled in the DMP in the past. Both, assessment of selection bias and analysis of outcome quality showed similar results to our main analyses. We therefore suppose that there is a considerable difference between DMP and usual-care patients at baseline irrespective of a selection bias due to drop out.

After assessing the selection bias, we investigated selected outcome indicators to estimate the impact of the selection bias. DMP patients had a lower systolic BP at baseline (139 mmHg vs. 144 mmHg for usual-care patients) and DMP patients also showed a greater decrease in systolic BP between t_0 _and t_1_, that was 3.6 mmHg higher than for usual-care patients. The higher BP reduction for DMP patients starting from a better baseline value is difficult to interpret. On the one hand, it could be interpreted as evidence for the effectiveness of the DMP. On the other hand differences may be due to selection effects. In the analysis of the course of BP we controlled for the differences in BP at baseline in order to reduce the selection bias, but this procedure cannot be considered sufficient, because it is not possible to statistically control for the total selection effect. The selection bias is multifaceted and statistical adjustment cannot remove bias generated by factors that are unmeasured or imprecisely measured. For example, we do not know which measured or unmeasured factors (e.g. differences in compliance, nutrition or physical activity) may have led to the different BP values at baseline and to what extent these factors resulted in a greater decrease of systolic BP values in DMP patients during the time span of the study. Our conclusion therefore is that the existing selection bias of more active and motivated DMP patients with a lower risk for diabetic complications makes it impossible to decide if the DMP is responsible for the differences in the course of BP between DMP and usual-care patients.

### Strengths and weaknesses of the study

Our study is the first study on the German DMP with a baseline assessment before the individual start of the DMP. This is important because a baseline assessment is a precondition for estimating differences in patient characteristics between the comparison groups which could influence outcome quality. Another strength of our study is the long observation period with a mean time interval between t_0 _and t_1 _of 36 months.

We had an overall participation rate of 60% which is above the published rate of 36% to 42% [19-21] in other DMP studies. The participants in our study were not selected by the GP or patients' health care utilization patterns since we used a chart registry approach rather than a waiting room recruitment strategy. However, DMP and usual-care patients had different response rates, i.e. 70% of DMP and 46% of usual-care patients agreed to participate in the study. In part, this difference can be explained by the inferior patient documentation in usual-care patients including more invalid postal addresses for contact. The difference in response rates may also be a part of the DMP selection bias: In both groups the more active and motivated patients may also be more often interested in study participation. For this reason the number of usual-care patients with good self-management may be larger in our study than it is de facto. We thus may have even underestimated the selection bias.

Except for depression and hypertension, we did not collect standardized data on the patients' comorbidity. We showed that patients with a higher risk for diabetic complications are less likely to be in the DMP group. Analogously the presence of burdensome comorbidity might also keep patients from being enrolled into the program and may therefore also contribute to the selection bias. This hypothesis should be analysed in further research.

Interviewers were trained and monitored and we used double data entry with additional plausibility checks. It was not possible to get standardized clinical examinations of the study participants because of the retrospective assessment. Instead, we used laboratory data for GHb values and chart review data for the other variables. Other strengths of our study relate to a sufficient statistical power, multivariate analyses dealing with possible confounding and an advanced treatment of missing values.

We found considerable differences between imputed and available data. Against the background of a biased distribution of missing values (i.e. more missing values in usual-care patients), we interpret the differences in results when using available data as an effect of listwise deletion of subjects with missing values in the regression analyses. This bias was removed by multiple imputation. For this reason, we consider the results from the analyses of the imputed data as more valid.

### Comparison with other studies

Six studies on the effects of the German DMP on patients with type 2 diabetes have been published up to now. Two are observational studies based upon insurance claims data. One (BEK claims [22]) is cross-sectional whereas the other (ELSID claims [23]) is longitudinal but without baseline assessment. The four other studies are based on patient surveys, three of which are cross-sectional (BEK survey [19], ELSID survey [20] and KORA survey [24]) and one is longitudinal but also without baseline assessment (GMA survey [21]). Because of the missing baseline assessment in all studies mentioned above, none of these studies can distinguish between selection and program effects.

However, the results of some of these studies pointed at further dimensions of selection mechanisms in the DMP. The authors of the BEK survey found a higher level of education among DMP patients (e.g. 12% of DMP patients and 8.2% of usual-care patients have an university degree), explaining in part differences in health status between DMP and usual-care patients. Additionally there was a longer duration of diabetes among DMP patients [19]. In our study, we also found a longer duration of diabetes in DMP patients in a bivariate analysis, but after adjusting for confounders in the multivariate analysis, it did not contribute to the selection bias.

The BEK claims study focussed on comparisons of morbidity between DMP and usual-care patients. DMP patients were enrolled for 16 months on average. The authors found more severe comorbidity (i.e. myocardial infarction, heart failure, stroke as well as minor and major amputations) in usual-care patients and a higher rate of precursor diagnoses in DMP patients (i.e. angina pectoris and chronic ischemic heart disease). They interpreted these findings as evidence for a better outcome of DMP patients [22]. For methodical reasons, this interpretation is not conclusive in a cross-sectional study.

In the study presented here we could show that future DMP patients are more active and motivated already before enrolment. We therefore presume that DMP patients might have a better disease awareness and more frequently search medical advice. Additionally, a better medical surveillance is one of the main elements of the DMP. Both possibilities might increase the detection of precursor diagnoses, while highly severe diagnoses as myocardial infarction or stroke are normally identified independently of the patients' awareness and increased physicians' surveillance in ambulatory care. We therefore suggest that the results of the BEK claims study should be interpreted in the sense of a selection bias with a higher rate of highly severe diagnoses in usual-care patients.

## Conclusions

We conclude that self-active and motivated patients with a lower risk for diabetic complications seem to be more likely to be enrolled in the German DMP. The selection mechanisms of the DMP in part reflect the legal regulations. Policy makers explicitly defined that only active patients with potential benefit should be enrolled. This question touches aspects of appropriateness of care, equity and accessibility to healthcare. The major clinical implication of our results is that the disease management program probably does not reach a considerable amount of higher risk patients. In our view it is this patient subgroup that has the biggest need of assistance and the largest room for improvement. For this reason it may be important to develop the program further in the sense of especially enrolling higher risk patients. We are aware that this may be a strenuous exercise. Future research should focus on how to recruit this subgroup and how to promote adherence to such a program.

The systematic selection and drop out mechanisms in the DMP may lead to a bias in evaluation studies that consists of two elements:

• in studies that recruit patients after the intervention started, patients with inferior prognosis may have systematically dropped out of the intervention group before (retrospective) baseline assessment is performed, and

• in non-randomized studies a largely increased rate of patients with better prognosis may be in the intervention group because of specific enrolment criteria for the DMP.

Researchers who do not account for this bias may wrongly interpret their results as program effects while in fact they reflect the selection bias. The impact of the selection bias can be reduced if randomization is not possible. We suggest that future evaluation research should at least

• have a baseline assessment before the intervention takes place, so that the different starting points of DMP and usual-care patients can be taken into account, and

• ensure that all patients participating in the study should meet the criteria for DMP enrolment as applied in clinical reality, e.g. DMP and usual-care patients in the study should have the same risk status as well as comparable levels of motivation and activity.

## Competing interests

The authors declare that they have no competing interests.

## Authors' contributions

HK, CK, IS and HvdB designed the project. CK and BG supervised the data collection. IS analysed the data and drafted the manuscript. AT performed the power calculation and gave statistical advice. HK, CK, ECvL, KW and HvdB reviewed the different phases of the manuscript. All authors read and approved the final manuscript.

## Pre-publication history

The pre-publication history for this paper can be accessed here:

http://www.biomedcentral.com/1472-6963/10/351/prepub
